# Perioperative Anaphylaxis in Japanese Secondary Care Institutions: Incidence, Causes, and the Imperative for Improved Diagnostic Practices

**DOI:** 10.7759/cureus.57555

**Published:** 2024-04-03

**Authors:** Yasuyuki Suzuki, Shuang Liu, Natsumi Yamashita, Naohito Yamaguchi, Yasushi Takasaki, Toshihiro Yorozuya, Masaki Mogi

**Affiliations:** 1 Anesthesiology, Saiseikai Matsuyama Hospital, Matsuyama, JPN; 2 Research Division, Saiseikai Research Institute of Health Care and Welfare, Tokyo, JPN; 3 Department of Pharmacology, Ehime University Graduate School of Medicine, Toon, JPN; 4 Clinical Research Center, National Hospital Organization Shikoku Cancer Center, Matsuyama, JPN; 5 Department of Anesthesia and Perioperative Medicine, Ehime University Graduate School of Medicine, Toon, JPN

**Keywords:** drug hypersensitivity reaction, shock, allergy, anesthesia, anaphylaxis

## Abstract

Background

This research investigates the incidence, suspected causes, and diagnostic procedures for perioperative anaphylaxis (POA), a potentially severe complication, in secondary care hospitals across Japan.

Methodology

We surveyed Saiseikai hospitals and gathered data on surgical procedures, POA occurrences, potential triggers, and diagnostic methods.

Results

Among 70,523 surgeries, seven were associated with POA, resulting in an approximate incidence rate of 0.01%. Rocuronium was the most commonly suspected trigger, followed by sugammadex, latex, and angiography contrast agents. Despite the importance of skin tests as the most basic and crucial diagnostic method, they were conducted in only three instances. No in vitro tests for drug identification were conducted, and in four cases, the cause was determined merely based on the timing of drug administration, indicating significant diagnostic limitations.

Conclusions

The study underscores the critical situation in Japan regarding insufficient diagnostic practices and difficulties in identifying triggering drugs rather than the consistent prevalence of POA in secondary care facilities. The findings emphasize the need for improved diagnostic proficiency and more rigorous drug identification practices to ensure prompt and accurate POA diagnosis. It is essential to conduct further research and interventions to increase patient safety during the perioperative period in secondary care settings.

## Introduction

Perioperative anaphylaxis (POA) is a life-threatening complication, and understanding its overall picture is crucial for anesthesiologists. However, investigating this condition is challenging due to its extremely low incidence of approximately 1 in 10,000 cases [1-3]. Moreover, the anesthetic methods and medications vary by era and among countries and regions. Therefore, grasping the latest incidence status by region and country is an important research topic for providing safe anesthesia management.

In 2023, Takazawa et al. conducted a large-scale survey on POA, focusing on tertiary medical institutions, such as university hospitals in Japan, and reported their findings [4,5]. However, in Japan, surgeries are still frequently performed in small and medium-sized hospitals with around 200 beds. Large-scale hospitals tend to perform highly advanced surgeries, including transplant surgeries, and the patient background and medications used may differ from those in small and medium-sized hospitals.

The first author (Y.S.) belongs to the Social Welfare Organization Saiseikai Imperial Gift Foundation, Inc., a medical organization established by Emperor Meiji in 1911. The Saiseikai aims to provide adequate medical care to the impoverished and continues to support regional healthcare by actively providing medical services to public assistance recipients while continuing general medical care. The Saiseikai organization, through its affiliated tertiary and secondary care facilities, serves a wide range of patients within the regions where these hospitals are located. This study included patients who underwent surgery at hospitals affiliated with the Social Welfare Organization Saiseikai Imperial Gift Foundation, Inc. No patients from other hospitals or healthcare systems were included.

Investigating POA in the Saiseikai hospital group is significant because the group comprises hospitals of various scales, from large tertiary care centers to smaller secondary care facilities, and serves a wide range of patients across different regions of Japan. This diversity allows for a more comprehensive assessment of POA incidence and management in regional healthcare settings, which may differ from that in highly specialized academic centers. Furthermore, the findings from this study may be more generalizable to the overall medical situation in Japan compared to studies focusing on a single institution or a narrow subset of hospitals.

This article was previously posted to the medRxiv preprint server on November 8, 2022 [6].

## Materials and methods

We conducted a questionnaire survey of 56 Saiseikai-affiliated hospitals with inpatient beds, asking them to report POA cases that occurred between January 2021 and December 2021. To ensure data quality, we provided clear instructions and definitions to the respondents and reviewed the submitted data for inconsistencies or errors, following up with hospitals for clarification when necessary. While these measures cannot guarantee the absolute validity of the data, they help mitigate the risk of inaccuracies inherent in questionnaire-based studies. We asked them to input their responses into Google Forms. The survey asked hospitals to retrospectively report POA cases that had occurred during the specified one-year period. We did not conduct a separate retrospective investigation beyond the survey itself. The diagnostic criteria for anaphylaxis were based on the “Anaphylaxis Guidelines 2021” published by the Japanese Society of Allergology (JSA) (Appendix 1). These guidelines are widely recognized and adopted by healthcare professionals in Japan and are publicly available on the JSA website (https://anaphylaxis-guideline.jp/). While we cannot guarantee that every institution surveyed strictly adhered to these guidelines, their widespread acceptance within the Japanese medical community makes them a suitable standard for our study.

Based on the specified criteria, an anaphylaxis diagnosis hinges on the manifestation of rapidly evolving cutaneous or mucosal symptoms, such as widespread urticaria or significant swelling of the lips and tongue (Appendix 1). This diagnosis is further supported when these symptoms are accompanied by at least one of the following additional signs: respiratory issues including dyspnea, hypoxemia, or wheezing; cardiovascular disturbances such as syncope, incontinence, or altered states of consciousness; or gastrointestinal problems characterized by intense abdominal pain or repeated vomiting.

It is important to note that a diagnosis of anaphylaxis can be considered even in the absence of the hallmark cutaneous or mucosal manifestations, provided there is a history of ingestion or exposure to a known allergen likely to be the trigger. This highlights the variability in the presentation of anaphylaxis and the importance of a comprehensive diagnostic approach accounting for both the clinical symptoms and the patient’s allergen exposure history.

It should be noted that the JSA revised its diagnostic guidelines for anaphylaxis in August 2022 [7] after the completion of our study. While the updated guidelines introduce some changes to the diagnostic criteria, they do not have a direct impact on the interpretation of our findings, as our study was conducted using the previously established JSA guidelines that were in effect during our data collection period. Nevertheless, we acknowledge the importance of staying informed about the latest developments in anaphylaxis diagnosis and management, and future research should consider the implications of the revised guidelines. The survey items in our study included the total number of surgeries, the number of surgeries under general anesthesia, the number of anaphylaxis cases, the tests performed for diagnostic assistance, and the suspected causative drugs. This study was conducted with the approval of the Ethics Committee of the Saiseikai Research Institute of Health and Welfare (approval number: 2022-01). However, a significant limitation is that we focused on investigating the incidence and did not plan a detailed case-by-case examination, which the Ethics Committee approved.

To visualize the difference in the incidence of POA between urban and rural areas, we plotted the medical institutions where anaphylaxis occurred as circles and those where it did not occur as triangles on a map of Japan. The visualization was performed using the ggmap package version 3.0.0 on R version 4.1.3.

Moreover, to investigate the influence of hospital size (number of beds) and the number of surgeries on the incidence of POA, we created a scatter plot with the number of beds on the X-axis and the number of surgeries on the Y-axis, providing a visual representation of the relationship between these factors and POA occurrence. Facilities where POA occurred were plotted as circles, and those where it did not occur were plotted as triangles. We calculated the incidence by dividing the facilities into high-volume centers with many surgeries and low-volume centers with fewer surgeries. Fisher’s exact test was used to determine if there was a significant difference between the two groups.

As the incidence of POA may be influenced by the total number of surgeries, we set a 95% confidence interval based on the Poisson distribution. We drew a funnel plot to evaluate the incidence more accurately. For plotting, we modified an R script published by Dr. Fukui (https://fukui-ke-0507.shinyapps.io/inc_funnel/) with his permission. The libraries used were FunnelPlotR, COUNT, and ggplot2.

## Results

Among the 56 medical institutions, 35 facilities responded to the survey (Tables 1, 2). One facility was excluded because it did not perform surgeries, and data from 34 facilities were used for analysis. The median number of beds in the responding hospitals was 243 (interquartile range (IQR) = 199-397.5), while the median number of beds in the non-responding hospitals was 248 (IQR = 128.5-376). The Wilcoxon rank-sum test showed no significant difference in the number of beds between the responding and non-responding hospitals (W = 956.5, p = 0.1328) (Appendix 2). The total number of surgeries was 70,523, and seven cases were diagnosed with POA, resulting in an incidence of 0.010%.

**Table 1:** List of healthcare organizations that cooperated with our survey.

List of healthcare organizations that cooperated with our survey
Saiseikai Karatsu Hospital, Saga, Japan
Isikawaken Saiseikai Kanazawa Hospital, Ishikawa, Japan
Saiseikai Toyama Hospital, Toyama, Japan
Higasikanagawa Rehabilitation Hospital, Kanagawa, Japan
Saiseikai Fukuoka General Hospital, Fukuoka, Japan
Saiseikai Misumi Hospital, Kumamoto, Japan
Saiseikai Kanagawa Hospital, Kanagawa, Japan
Saiseikai Niigata Hospital, Niigata, Japan
Saiseikai Yokohamashi Nanbu Hospital, Kanagawa, Japan
Saiseikai Gose Hospital, Nara, Japan
Okayama Saiseikai general Hospital, Okayama, Japan
Kitakami Saiseikai Hospital, Iwate, Japan
Saiseikai Nara Hospital, Nara, Japan
Kagawaken Saiseikai Hospital, Kagawa, Japan
Saiseikai Nakatsu Hospital, Osaka, Japan
Fukuiken Saiseikai Hospital, Fukui, Japan
Ryugasaki Saiseikai Hospital, Tochigi, Japan
Saiseikai Kumamoto Hospital, Kumamoto, Japan
Saiseikai Wakayama Hospital, Wakayama, Japan
Saiseikai Otaru Hospital, Hokkaido, Japan
Saiseikai Arita Hospital, Wakayama, Japan
Niigataken Saiseikai Sanjo Hospital, Niigata, Japan
Yamagutiken Saiseikai toyoura Hospital, Toyama, Japan
Saiseikai Fukusima General Hospital, Fukushima, Japan
Saiseikai Iizuka Kaho Hospital, Fukuoka, Japan
Saiseikai Yokohamasi Toubu Hospital, Kanagawa, Japan
Saiseikai Imabari Hospital, Ehime, Japan
Saiseikai Yahata General Hospital, Fukuoka, Japan
Oita Prefecture Saiseikai Hita Hospita, Oita, Japan
Saiseikai Sendai Hospital, Miyagi, Japan
Shimaneken Saiseikai Gotu General Hospital, Shimane, Japan
Tokyo Saiseikai Central Hospital, Tokyo, Japan
Saiseikai Kyoto Hospital, Kyoto, Japan
Saiseikai Moriyama Municipal Hospital, Shiga, Japan
Saiseikai Matuyama Hospital, Ehime, Japan

**Table 2:** Table showing the occurrence of anaphylaxis in each case.

Hospital	Number of operations	Number of beds	Number of anaphylaxis
Kyushu			
S_01	1,074	193	0
S_04	3,941	390	2
S_05	118	128	0
S_17	4,311	400	0
S_24	47	197	0
S_27	1,833	399	0
S_28	899	199	1
S_29	1,229	244	0
Hokuriku			
S_02	1,085	260	0
S_03	2,354	250	0
S_07	4,114	425	0
S_15	5,058	460	0
S_21	814	199	0
Kanto			
S_06	1,703	199	0
S_08	4,549	500	1
S_16	887	210	1
S_25	6,056	562	0
S_31	4,361	535	1
Kansai			
S_09	1,020	167	0
S_12	1,061	194	1
S_14	5,107	670	0
S_18	1,100	200	0
S_20	1,195	184	0
S_32	1,625	288	0
S_33	190	199	0
Chugoku			
S_10	6,229	473	0
S_22	489	275	0
S_30	68	280	0
Tohoku			
S_11	1,415	224	0
S_23	450	216	0
Shikoku			
S_13	1,616	198	0
S_26	1,461	191	0
S_34	1,265	199	0
Hokkaido			
S_19	1,433	378	0

**Figure 1:**
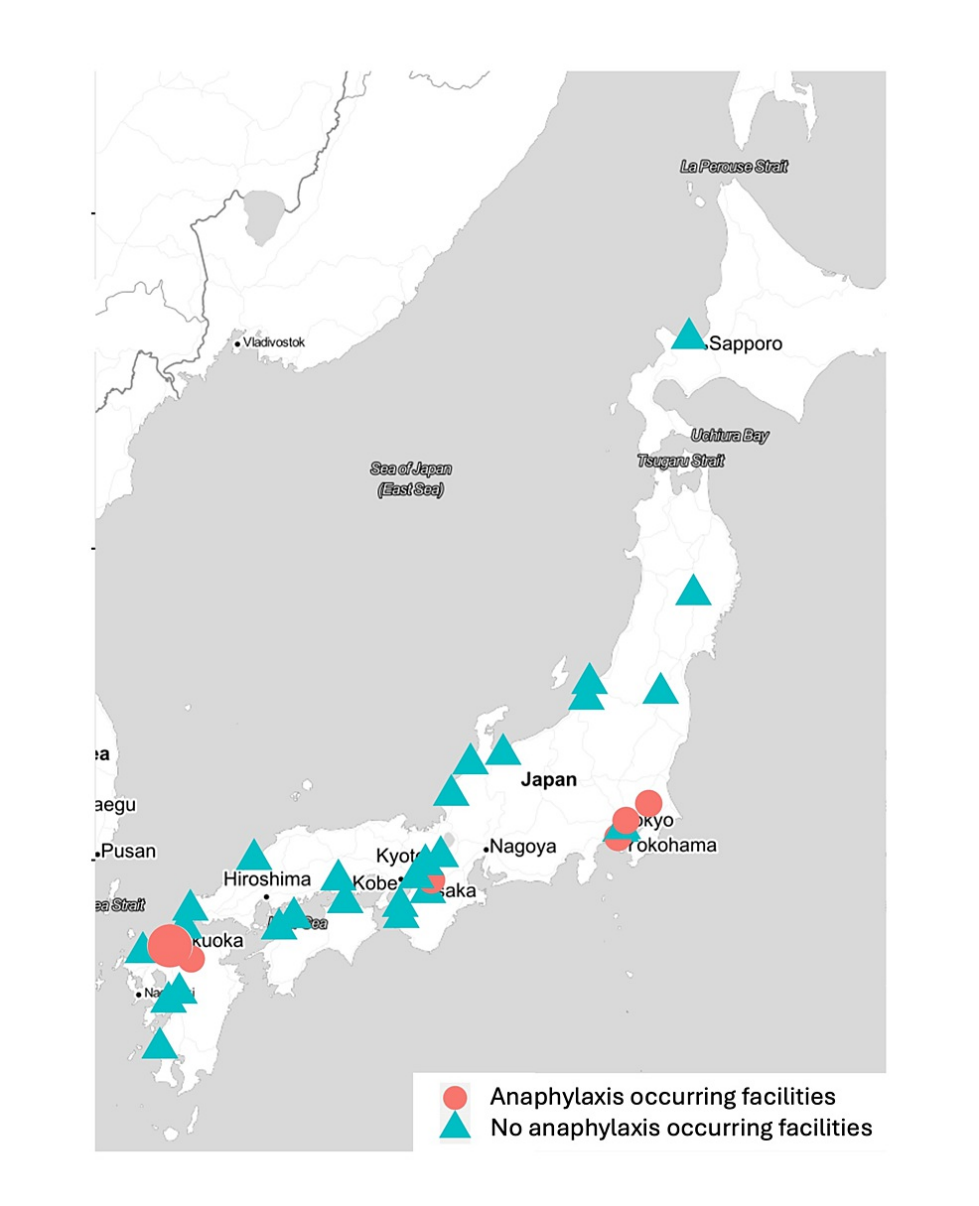
Location distribution of facilities where perioperative anaphylaxis occurred. The healthcare facilities surveyed are distributed not only in urban areas but also in rural areas. Facilities experiencing perioperative anaphylaxis are concentrated in Kanto (e.g., Tokyo), Kansai (e.g., Osaka), and Kitakyushu (e.g., Hakata) regions. These regions have high population densities and witness more surgeries and, consequently, more cases of POA. The map was created using the ggmap package version 3.0.0 on R version 4.1.3. Red circles: facilities where anaphylaxis occurred. Blue triangle: facilities that did not report anaphylaxis occurrence.

Medical institutions where POA occurred and those where it did not occur were plotted on a map of Japan to visualize the incidence difference between urban and rural areas. The results showed that POA was only distributed in the Kanto, Kansai, and North Kyushu regions, among Japan’s most densely populated areas (Figure 1).

**Figure 2:**
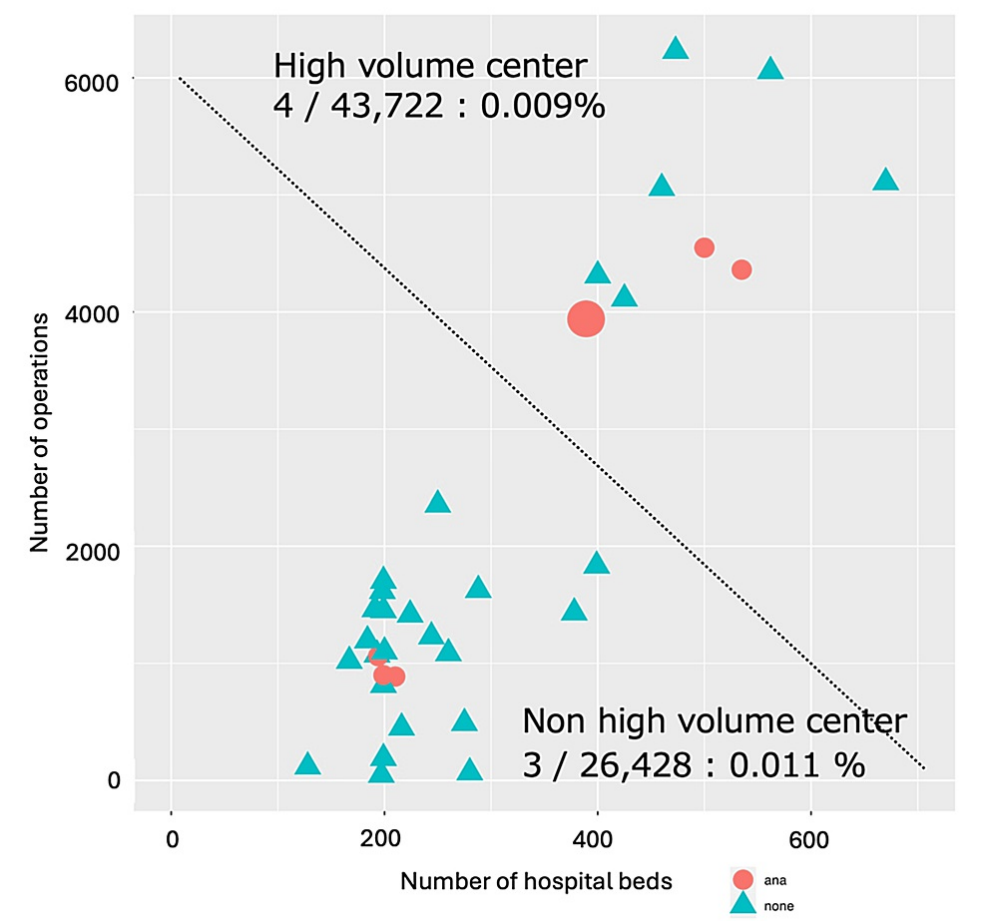
The association between the occurrence of perioperative anaphylaxis and the number of beds and surgeries per facility. Scatter plots visualize whether anaphylaxis is more common in high-volume centers with many operations than in non-high-volume centers. The incidence of both groups was examined using Fisher’s exact test, which showed no significant difference (p = 1.00). Scatterplot analysis and Fisher’s exact test were performed using R version 4.1.3. Red circles represent facilities where anaphylaxis occurred. Blue triangles represent facilities that did not report anaphylaxis occurrence.

The incidence of POA was compared between high-volume centers with many surgeries and low-volume centers with fewer surgeries. The incidence was 0.011% in high-volume centers and 0.009% in low-volume centers. Fisher’s exact test showed no statistically significant difference, with a p-value of 1.00 (Figure 2).

**Figure 3:**
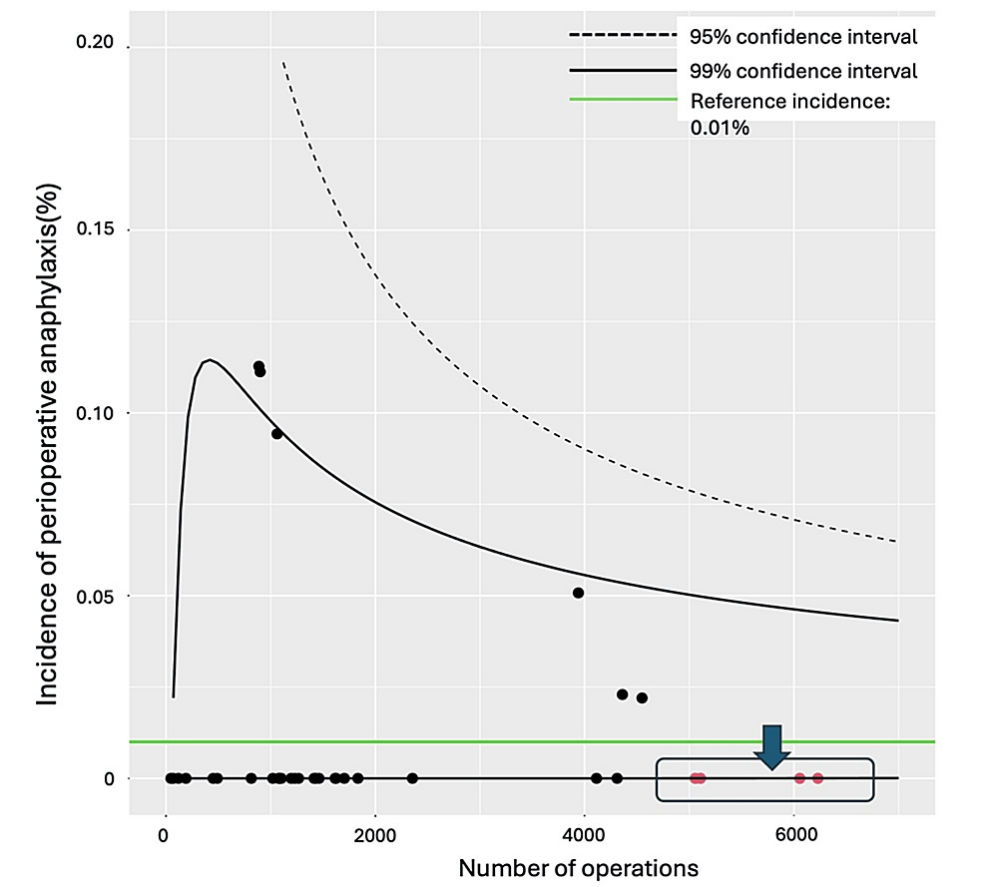
Funnel plot analysis to determine the association between the number of surgeries and the incidence of anaphylaxis. The funnel plot visually represents the incidence of perioperative anaphylaxis across various medical facilities concerning the number of surgeries performed. Each point on the plot corresponds to a specific medical facility, with its position indicating the number of surgeries conducted and the observed incidence of perioperative anaphylaxis. The central line represents an expected incidence rate of 0.01%. The outer lines delineate the 95% confidence interval, indicating where the incidence rates for most facilities are expected to fall if there is no significant deviation from the expected rate. The four facilities contained within the square indicated by the blue arrow, while falling outside the confidence interval, have a high volume of surgeries and likely experienced no anaphylaxis events by chance alone. Therefore, this deviation is not considered clinically significant.

In the funnel plot analysis with a POA incidence set at 0.01%, 30 facilities fell within the 95% confidence interval. In contrast, four facilities with many surgeries deviated from the confidence interval (Figure 3).

The tests used to aid in diagnosing anaphylaxis were intradermal tests in three cases and quantification of histamine and tryptase in one case. The remaining three cases were diagnosed based on clinical symptoms alone.

The presumed causative drugs were rocuronium in three cases, sugammadex in two cases, and latex and angiographic contrast agent in one case each. In the two cases of rocuronium-induced anaphylaxis and the case of sugammadex-induced anaphylaxis, the causative drug was confirmed by intradermal tests. For the other reported cases, where intradermal tests were not performed, the attending anesthesiologists and allergy specialists at each participating hospital assessed the most likely causative agent based on the temporal relationship between drug administration and the onset of symptoms. However, we acknowledge that this method of attribution is less definitive than confirmatory intradermal testing and may be subject to interpretation bias.

## Discussion

The reported incidence of POA in the Saiseikai hospital group was 0.010%, which is similar to the rates reported in several previous studies [1-3]. However, it is important to note that this incidence is based on clinical diagnoses made by the participating hospitals and not on definitive diagnoses confirmed by skin tests or other diagnostic procedures. In fact, we found that skin tests, which are considered the gold standard for identifying causative agents in POA, were performed in only three out of the seven reported cases. This low rate of confirmatory testing, along with the lack of standardized diagnostic criteria, raises concerns about the potential for misdiagnosis or overdiagnosis of POA in our study. These limitations may affect the accuracy of our findings and should be considered when interpreting our results. Our study highlights the need for the development and implementation of standardized diagnostic protocols for POA, including the use of skin tests or other confirmatory tests whenever possible, to improve the accuracy and reliability of future research in this area.

The National Audit Projects 6 (NAP6) is the most extensive study on POA, reporting an incidence of 1 in 10,000 cases [2]. In Japan, Takazawa et al. reported an incidence of 0.036% in tertiary medical institutions [3]. These values are consistent with our survey results, suggesting that the influence of country, region, or hospital size is minimal. Furthermore, it implies that safe perioperative management is being performed in many medical institutions, which is a favorable outcome for patients undergoing surgery.

In our survey, we found that rocuronium was the most commonly reported causative agent for POA, which is similar to the findings of Takazawa et al. [4]. This may be partly explained by the fact that rocuronium is the only non-depolarizing neuromuscular blocking agent available in Japan. We also noted two cases of anaphylaxis attributed to sugammadex out of the seven total POA cases reported. While sugammadex has been associated with anaphylaxis in previous reports from Japan [4,8], the limited number of cases in our study precludes a direct comparison of incidence rates. Further research with larger sample sizes is needed to establish the consistency of our findings with other studies and better understand the role of sugammadex in POA in Japan.

According to the Food and Drug Administration (FDA) summary review [9], hypersensitivity reactions, including one case of anaphylaxis, were reported in clinical trials of sugammadex. The review underscores the crucial need for anesthesiologists to carefully consider the potential for hypersensitivity reactions when administering this drug.

Sugammadex is a substance with a molecular weight of 2,178, having a cyclodextrin structure with side chains modified to fit the molecular structure of rocuronium. Substances with a cyclodextrin structure are widely used as deodorizers in daily life. It has been reported that using cosmetics containing quaternary ammonium compounds as additives can cause hypersensitivity reactions to rocuronium [10]. This is thought to be due to the production of specific IgE antibodies that cause cross-reactivity due to sensitization in daily life. POA due to sugammadex may occur through a similar mechanism. Additionally, sugammadex’s more significant molecular weight compared to other drugs used perioperatively may be one factor in facilitating sensitization [11]. As far as the FDA is concerned, it is essential to consider the possibility of hypersensitivity reactions when using sugammadex.

Hypersensitivity reactions to contrast agents occur through the Mas-related G protein-coupled receptor X2 pathway, which can occur even without sensitization [12,13]. It has been suggested that reactions via this receptor can also occur with neuromuscular blocking agents and antibiotics, which is a point that should be continuously focused on in POA research.

The most important point from this study’s results that should be translated into clinical practice is that skin tests, the gold standard for identifying the causative drug, were performed in only three out of seven cases. Although some guidelines suggest that a diagnosis can be made based on clinical symptoms alone [14], we strongly recommend establishing a system that facilitates the implementation of diagnostic tests, particularly skin tests, to provide a more definitive diagnosis. Skin tests are considered the gold standard for identifying causative drugs in POA, and although they may require additional costs for acquiring suspected allergens and monitoring for adverse reactions, they are likely to be more cost-effective than other diagnostic tests, such as in vitro assays. We believe that enabling anesthesiologists to perform skin tests themselves could be an efficient and cost-effective approach to improving the diagnosis and management of POA. However, further research, including a formal cost-benefit analysis, is needed to confirm this hypothesis and guide the implementation of such a system.

The NAP6 states that identifying the causative drug is crucial for patients who have experienced POA to receive safe medical care in the future [2]. To achieve this, a combination of multiple tests should be used, as all tests have the possibility of false negatives.

The ImmunoCAP method is well known as a specific IgE antibody test, but it is costly and often results in false negatives, even in anaphylaxis cases [15,16]. The basophil activation test, which has recently gained attention, separates basophils from the patient’s blood, reacts with the suspected drug, and observes changes in cell surface markers [17,18]. It was also used as a central test in the large-scale study by Takazawa et al. [4]. However, the activity may decrease within a few hours after blood collection, and it is recommended that the test is performed within 24 hours. Additionally, flow cytometry is required, which is a challenge as it can only be performed in research institutions such as universities. The mast cell activation test using patient serum does not have a time limit after blood collection, but securing cultured mast cells that show stable reactions is problematic [18-20].

Skin tests can cause severe hypersensitivity reactions, albeit extremely rarely. However, they are a safe and inexpensive test with a lower false-negative rate than other tests [21]. Therefore, it is the most basic and essential test for identifying the causative drug in anaphylaxis. On the other hand, specialized knowledge, such as setting the dilution series of the suspected drug, is required. As dermatologists usually perform it, it is a test with a high hurdle for anesthesiologists. However, anesthesiologists have the skills to respond to sudden changes due to hypersensitivity reactions during skin tests. In the future, this problem may be solved by anesthesiologists actively performing skin tests.

Finally, we acknowledge several limitations of our study. First, the use of a questionnaire survey may introduce potential inaccuracies in reporting and does not allow for verification of strict adherence to diagnostic guidelines. Although we provided the JSA anaphylaxis guidelines as a reference to the participating hospitals, we did not collect detailed information on how these guidelines were interpreted and applied by individual clinicians in each case. As a result, we cannot definitively ensure that all reported cases of anaphylaxis were diagnosed using consistent criteria across the participating hospitals, which may affect the reliability and comparability of our findings.

Moreover, given the challenges of conducting a rigorous, prospective study across multiple institutions, our approach, while valuable in providing insights into the incidence and characteristics of POA in Japanese hospitals, has inherent limitations. Future studies using more robust methodologies and capturing more granular data on the specific diagnostic criteria used in each case may help to confirm and extend our findings.

## Conclusions

Our study found that the incidence of POA in the Saiseikai hospital group was consistent with previous reports, but diagnostic confirmation was performed in only a limited number of cases. This highlights the need for improved implementation of standardized diagnostic protocols, particularly skin tests, to identify causative agents and ensure patient safety. We strongly recommend prioritizing definitive diagnosis in all suspected POA cases. Future studies should focus on developing and promoting standardized guidelines to reduce the incidence and severity of POA in Japan.

## Figures and Tables

**Figure 4:**
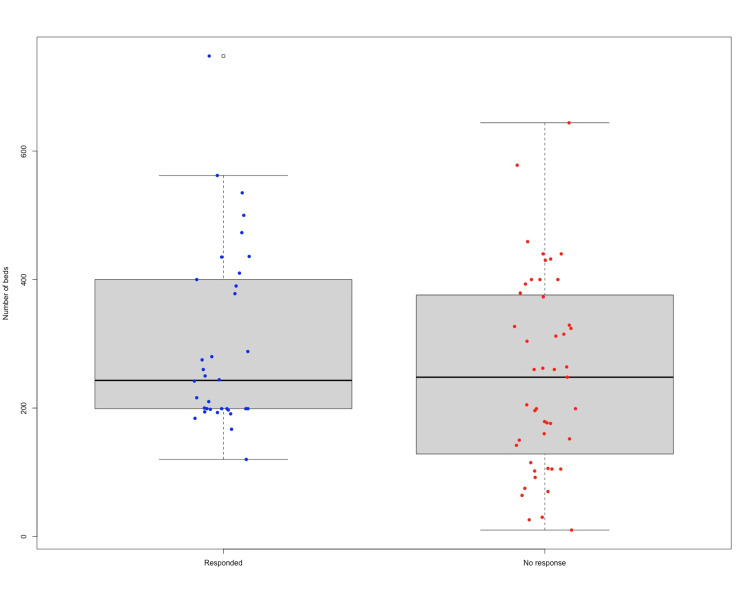
Box plot comparing the number of beds in responding and non-responding hospitals. The median number of beds in the responding hospitals was 243 (interquartile range (IQR) = 199-397.5), while the median number of beds in the non-responding hospitals was 248 (IQR = 128.5-376). The Wilcoxon rank-sum test showed no significant difference in the number of beds between the responding and non-responding hospitals (W = 956.5, p = 0.1328).

## Appendices

Appendix 1

**Table 3:** Diagnostic criteria for anaphylaxis based on the Japanese Society of Allergology (JSA) guidelines. The diagnostic criteria for anaphylaxis used in this study were derived from the JSA guidelines. The criteria consist of two main components: Rapidly progressing cutaneous or mucosal symptoms, accompanied by at least one of the following: (a) respiratory symptoms, (b) cardiovascular symptoms, and (c) gastrointestinal symptoms. In the absence of typical cutaneous or mucosal symptoms, anaphylaxis can be diagnosed if the patient was exposed to a likely allergen and rapidly developed any of the following: (a) hypotension, (b) bronchospasm, and (c) laryngeal symptoms.

1. Rapidly progressing cutaneous or mucosal symptoms (such as generalized urticaria or swelling of the lips and tongue), accompanied by at least one of the following:
a. Respiratory symptoms (such as dyspnea, hypoxemia, or wheezing)
b. Cardiovascular symptoms (such as syncope, incontinence, or consciousness disturbance)
c. Gastrointestinal symptoms (such as severe abdominal pain or recurrent vomiting)
2. Even in the absence of typical cutaneous or mucosal symptoms, if the patient ingested (or was exposed to) an allergen with a high likelihood of being the cause and rapidly developed any of the following:
a. Hypotension
b. Bronchospasm
c. Laryngeal symptoms

Appendix 2
